# The Effect of Weather and Location of Fruit within the Tree on the Incidence and Severity of Citrus Black Spot on Fruit

**DOI:** 10.1038/s41598-020-58188-z

**Published:** 2020-01-29

**Authors:** Katherine E. Hendricks, Mary C. Christman, Pamela D. Roberts

**Affiliations:** 1 University of Florida, IFAS-Southwest Florida Research and Education Center, Department of Plant Pathology, Immokalee, FL 34142 USA; 2 University of Florida, Department of Statistics, Gainesville, Florida 32611 USA

**Keywords:** Plant sciences, Ecological epidemiology

## Abstract

Citrus black spot (CBS) caused by the fungus *Phyllosticta citricarpa* occurs in tropical and sub-tropical citrus production regions and affects all varieties of citrus. In Florida, the disease cycle is unique, having only the asexual spore. This work examines incidence and severity of CBS (hard spot symptoms) on fruit in two citrus groves during 2013–2014, 2014–2015 (Grove III) and 2015–2016 (Grove II) citrus seasons. Disease incidence and severity on fruit were analyzed based on citrus season, side of tree evaluated, height within the canopy, tree health, and tree age. Results indicate an increase in CBS incidence in Grove III between 2013–2014 and 2014–2015 seasons, with more infected or symptomatic fruit on the road side of the canopy and a higher incidence above 2 meters. Tree health status affected incidence but not severity and tree age had a significant effect on severity of CBS in Grove II. Analysis of weather data conducive for infection, between 2010 and 2017, indicated an average of 172 days per year (range: 104–261 days) when the temperature (15–35 °C) and relative humidity (RH ≥ 90% for 8 consecutive hours) were conducive for infection of fruit and an average of 98 days per year (range: 72–123 days) when the fruit were susceptible to infection.

## Introduction

Citrus black spot caused by the fungus *Phyllosticta citricarpa* was first discovered on sweet orange in southwest Florida in April 2010^1^. The disease has a worldwide distribution affecting all varieties of citrus within tropical and sub-tropical citrus production regions, particularly in warm, humid climates. The disease has been reported in Australia, South Africa^2^, and Argentina^3,4^ since the early 1900’s. It has more recently been introduced into Brazil, Cuba^5^, Uganda^6^, Ghana^7^, Italy, Malta, and Portugal^8^. The fungus invades and colonizes the fruit rind producing five distinct lesion types^9,10^. Lesions do not affect the internal quality of the fruit^10^, however in markets where fruit is quarantined it is precluded from sale in the fresh market, as opposed to endemic areas where less blemished fruit are sold. Fruit symptoms include hard spots (also known as shot hole spots), freckle spots, virulent spots, and false melanoses (see Supplementary Fig. S1). Interaction with spider mites produces cracked spots which have been observed in Brazil^11^ and Florida^1^. Yield losses due to premature fruit drop occurs under severe infections^7^. Florida’s citrus affected by this pathogen are placed under quarantine by the United States Department of Agriculture, restricting interstate movement of citrus materials. This places an additional economic cost due to the implementation of further phytosanitary practices in order to treat and move material within and outside of the state and country. Since the initial find in 2010, the quarantine zone has been extended to encompass 1160.32 km^2^ (286,720 acres) within Lee, Charlotte, Collier, Hendry and Polk counties as of July 2019^12^.

The life cycle of *P. citricarpa* consists of a sexual (ascospores) and asexual (conidia) phase. The fungus forms latent infection in citrus tissues. Leaves produce ascospores within 40 to 180 days of leaf fall^2,10^ depending on environmental conditions. Leaves, twigs, fruit, and thorns produce conidia. Rainfall, irrigation, or heavy dew is sufficient to trigger the release of spores - ascospores from mature asci are ejected into the air and dispersed by wind currents, constituting long distance travel, and gelatinous masses of conidia are splashed dispersed over shorter distances. Fruit are susceptible to infection for up to 6 months (24 weeks) after petal fall^13^ and leaves up to 10 months from flush^14,15^. Twig and thorn susceptibility windows have yet to be determined. Conidia have been considered less important than the ascospore in disease spread due to shorter dispersal distance by splash and reduced viability compared to the ascospore, however, recent work suggest that water and wind may disperse conidia over longer distances than previously reported^16^.

Both the sexual and asexual spores of CBS are present on citrus^17^ with the exception of Florida. In Florida, only one mating type has been found^17,18^ and genomic analysis using 15 simple sequence repeat markers for *P. citricarpa* revealed a clonal population^17^. In Brazil, the conidia have been shown to be a major inoculum source in infected groves^19^. In Florida, the conidia appear to be the sole spore type spreading the disease. Florida isolates produce conidia *in vitro* between 15 and 27 °C, with time to emergence between 24 to 14 days^20^. Brazilian isolates of *P. citricarpa* germinate and form appresoria at temperatures between 10 and 40 °C when exposed to moisture for 48 hours^21^. Following storage of citrus fruit at 4.5 and 10 °C, conidia can remain viable^22^ and approximately 40% percent will remain viable at 25 °C for up to 4 days, but are 100% non-viable at 30 days^9^.

The majority of the literature attributes the incidence and severity of CBS on fruit to a combination of ascospore and conidia inocula. The situation in Florida gives a unique opportunity to study the incidence and severity of CBS hard spot lesions in a system lacking ascospores. This work examines the incidence and severity of CBS on fruit spatially distributed within the tree canopy over a 3-year period (2013/14 to 2015/16) in one grove with 100% tree incidence of CBS at the beginning of the study and for a single citrus season (2015/16) in a second grove with 26.6% tree incidence.

## Results

### Weather and susceptibility

The number of days for fruit susceptibility with appropriate weather conditions for infection by conidia of *P. citricarpa* is given in Table 1. Defining fruit susceptibility as days with temperatures between 15 and 30 °C and a total daily rainfall ≥0.25 mm, with fruit on the trees within the first 24 weeks post fruit set (wpfs), IN3 (IN = potential infection period) gave consistently higher number of susceptible days than IN1, IN2, IN4 or IN5, with the exception of 2013, when there were more days with RH ≥ 90% (Table 1). Since the buildup of inoculum in the grove is an important factor to consider, we looked at the weather conditions conducive for conidial infection across the entire year, defining IN6 to IN10 as weather conditions conducive to infection of citrus tissue other than the fruit. Overall there were more days throughout the years when conditions were conducive for the infection of citrus tissue defined as days with temperatures between 15 and 30 °C and RH ≥ 90% in all years examined, especially in 2013 where 261 days out of the year were conducive to tissue infection based on this definition (Table 1).

**Table 1:** Weather and Susceptibility Data associated with ‘Valencia’ orange groves surveyed in Florida for citrus black spot caused by *Phyllosticta citricarpa*.

Year	Fruit Susceptible Days^a^	FRUIT SUSCEPTABILITY (DAYS)					Year	TREE TISSUE SUSCEPTABILITY (DAYS)				
		IN1^b^	IN2^c^	IN3^d^	IN4^e^	IN5^f^		IN6^g^	IN7^h^	IN8^i^	IN9^j^	IN10^k^
2010	3/25–9/27	8	39	88	32	6	2010	38	104	127	53	13
2011	2/23–9/19	22	44	72	25	2	2011	79	149	104	51	17
2012	2/15–9/14	20	56	79	30	7	2012	71	160	121	62	23
2013	2/6–9/25	33	160	108	94	21	2013	82	261	129	113	29
2014	3/29–10/30	29	84	110	49	16	2014	96	190	153	86	36
2015	2/17–9/30	35	90	123	69	25	2015	76	185	167	106	44
2016	4/4–10/31	9	62	108	42	3	2016	25	159	149	70	5
2017*	2/20–9/26	5	32	23	12	0	2017*	25	70	32	20	3

^a^Fruit Susceptible Days are the number of days when fruit are on the tree and are considered susceptible to infection by *Phyllosticta citricarpa* (between fruit set and 24 weeks post fruit set). Dates represent the estimated date of earliest fruit set and latest date of susceptibility at 24 weeks post fruit set. Temperature (T), Relative Humidity (RH) and Dew Point (DP) are based on occurring within the selected parameter for *a minimum of 8 consecutive hours.*

^b^IN1 = T_15–35 °C_ + DP ≥ T_min_ for 8 hr + Susceptible Fruit.

^c^IN2 = T_15–35 °C_ + RH ≥ 90% for 8 hr + Susceptible Fruit.

^d^IN3 = T_15–35 °C_ + TDR ≥ 0.25 mm + Susceptible Fruit.

^e^IN4 = T_15–35 °C_ + RH ≥ 90% for 8 hr + TDR ≥ 0.25 mm + Susceptible Fruit.

^f^IN5 = T_15–35 °C_ + RH ≥ 90% for 8 hr + TDR ≥ 0.25 mm + DP ≤ T for 8 hr + Susceptible Fruit.

^g^IN6_ = _T_15–35 °C_ + DP ≥ T_min_ for 8 hr.

^h^IN7 = T_15–35 °C_ + RH ≥ 90% for 8 hr.

^i^IN8 = T_15–35 °C_ + TDR ≥ 0.25 mm.

^j^IN9 = T_15–35 °C_ + RH ≥ 90% for 8 hr + TDR ≥ 0.25 mm.

^k^IN10 = T_15–35 °C_ + RH ≥ 90% for 8 hr + TDR ≥ 0.25 mm + DP ≤ T for 8 hr.

### Effect of citrus season and fruit location (height and side) within the canopy on CBS incidence and severity in Grove III

**Figure 1:**
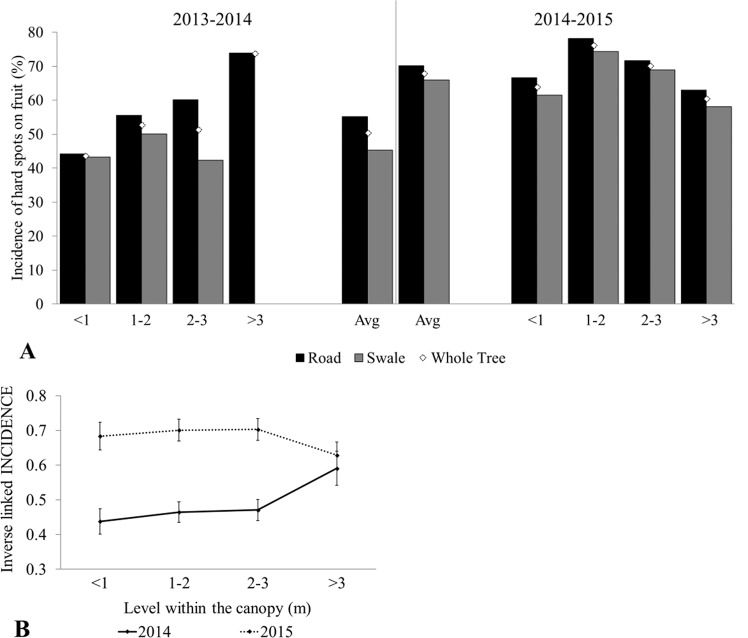
Incidence of Citrus Black Spot on fruit in Grove III for the 2013–2014 and 2014–2015 citrus season. (**A**) Citrus season and height of citrus black spot infected fruit within the canopy of ‘Valencia’ orange trees. (**B**) Least square means for the interaction between citrus season and height of citrus black spot infected fruit within the canopy with the 95% confidence interval.

Data on incidence and severity of hard spots on fruits are given in Table 2 and in Supplementary Table S1. Disease incidence was significantly different between citrus seasons (LSM ± SEM – 49.13 ± 2.92% in 2013–2014 vs. 67.96 ± 2.86% in 2014–2015 citrus season; P < 0.0001). Additionally, the overall incidence of CBS on fruit was significantly greater on the road side than the swale side of the tree, 61.49 ± 2.13% vs. 56.20 ± 2.44%, respectively (LSM ± SEM; Table 3), and there was a significant interaction between citrus season and the height within the canopy (P = 0.0041; Table 3). This interaction was due on average to a higher incidence of disease in the 2014–2015 citrus season than in the previous season, 2013–2014 season, while the average incidence of disease at the >3 m height was about the same between each season (Table 2, Fig. 1). Simple effect comparisons of the least square means of the interaction between citrus season and height within the canopy by citrus season revealed a statistical difference in disease incidence between <1 m vs. >3 m (43.81 ± 3.69% vs. 59.09 ± 4.90%, Adjusted P = 0.0129), 1–2 m vs. >3 m (46.49 ± 2.98% vs. 59.09 ± 4.90%, Adjusted P = 0.0408) but not 2–3 m vs. >3 m (47.09 ± 3.05% vs. 59.09 ± 4.90%, Adjusted P = 0.0590) during the 2013–2014 season. Similarly, no statistical difference (P > 0.5) was found in the different heights within the canopy during the 2014–2015 season.

**Table 2:** Grove III. Disease incidence and severity of hard spot lesions in the canopy during the 2013–2014 and 2014–2015 citrus season caused by *Phyllosticta citricarpa* in ‘Valencia’ oranges surveyed in Florida.

Citrus Season	Fruit Susceptible Days^a^	Assessment Date	Height within canopy	Disease Incidence (%) LSM ± SEM			Disease Severity Mean ± SE (%)^b^		
				Whole Tree	Road	Swale	Whole Tree	Road	Swale
2013–2014	[2013] 2/6–9/25	2/13 and 2/18/2014	Average	49.13 ± 2.92	52.08 ± 0.03	Non-est.	3.32 ± 0.22	3.73 ± 0.30	2.83 ± 0.32
			<1 m	43.81 ± 3.69^Aa^	47.20 ± 4.03	41.78 ± 4.71	2.75 ± 0.38	3.01 ± 0.53	2.50 ± 0.55
			1–2 m	46.49 ± 2.98^A^	46.75 ± 3.21	48.45 ± 3.76	3.55 ± 0.41	4.16 ± 0.59	2.95 ± 0.56
			2–3 m	47.09 ± 3.05 ^AB^	51.73 ± 3.53	43.58 ± 3.56	3.57 ± 0.37	4.04 ± 0.53	3.07 ± 0.53
			>3 m	59.09 ± 4.90^B^	62.39 ± 4.67	—	3.95 ± 0.87	3.95 ± 0.87	—
2014–2015	[2014] 3/29–10/30	3/31 and 4/1/2015	Average	67.96 ± 2.86	71.29 ± 0.03	64.32 ± 0.03	4.06 ± 0.20	4.11 ± 0.29	4.00 ± 0.28
			<1 m	68.36 ± 3.97^A^	72.95 ± 4.48	63.48 ± 4.94	3.80 ± 0.45	4.22 ± 0.71	3.39 ± 0.55
			1–2 m	70.05 ± 3.14^A^	73.60 ± 3.32	65.85 ± 4.01	4.57 ± 0.39	4.44 ± 0.54	4.70 ± 0.57
			2–3 m	70.28 ± 3.15^A^	72.49 ± 3.60	67.92 ± 3.93	4.24 ± 0.37	4.05 ± 0.46	4.43 ± 0.59
			>3 m	62.86 ± 3.83^A^	65.77 ± 4.50	59.82 ± 4.86	3.55 ± 0.37	3.68 ± 0.53	3.41 ± 0.51

^a^Fruit Susceptible Days are the date range when fruit are on the tree and are considered susceptible to infection by *Phyllosticta citricarpa* (between fruit set and 24 weeks post fruit set (wpfs)). Dates with year in brackets represent the estimated date of earliest fruit set and latest date of susceptibility at 24 wpfs.

^b^Severity assessments were based on a rating system devised by Sposito *et al*.^40^ ratings, ranging from 0.5% to 49% hard spot coverage on the visible surface of each fruit^28,40^. ^c^Disease Incidence for heights within the canopy for whole tree only – simple effect comparisons of the interaction between citrus season and height within the canopy LSM (±SEM) by citrus season. Least square mean values followed by the same letter are not significantly different (P > 0.05, Tukey-Kramer).

**Table 3:** Disease incidence and severity of citrus black spot symptoms (hard spots) on ‘Valencia’ oranges in Grove III (2013–2015). Effect of citrus season, side of the tree evaluated, height within the canopy and two-way interactions.

Fixed Effects	Variables		Disease Incidence (%) LSM ± SEM	Numerator Degrees of Freedom	Denominator Degrees of Freedom	F-Value	Pr > F
**Disease Incidence**							
Side of the tree evaluated	Road		61.49 ± 2.13	1	560.1	10.04	0.0016
	Swale		56.20 ± 2.44				
Citrus season	2013–2014		49.13 ± 2.92	1	301.3	20.10	<0.0001
	2014–2015		67.96 ± 2.86				
Height within the canopy	<1		56.48 ± 2.92	3	564.3	0.74	0.5312
	1–2		58.77 ± 2.32				
	2–3		59.20 ± 2.35				
	>3		60.99 ± 3.11				
Citrus season × Height within the canopy	2013–2014	<1	43.81 ± 3.69	3	564.5	4.47	0.0041
		1–2	46.49 ± 2.98				
		2–3	47.09 ± 3.05				
		>3	59.09 ± 4.90				
	2014–2015	<1	68.36 ± 3.97				
		1–2	70.05 ± 3.14				
		2–3	70.28 ± 3.15				
		>3	62.86 ± 3.83				
**Fixed Effects**	**Variables**		**Disease Severity**^**a**^ **(%) LSM ± SEM**	**Numerator Degrees of Freedom**	**Denominator Degrees of Freedom**	**F-Value**	**Pr > F**
**Disease Severity**							
Citrus season	2013–2014		1.56 ± 0.10	1	106	4.80	0.0306
	2014–2015		1.87 ± 0.10				
Side of the tree evaluated	Road		1.81 ± 0.08	1	106	5.38	0.0223
	Swale		1.64 ± 0.08				
Citrus season × Side	2013–2014	Road	1.73 ± 0.11	1	106	3.98	0.0486
		Swale	1.42 ± 0.11				
	2014–2015	Road	1.89 ± 0.11				
		Swale	1.86 ± 0.11				

^a^Severity assessments were based on the severity scale consisting of six levels, ranging from 0.5% to 49% hard spot coverage on the visible surface (exposed to sunlight) of the fruit, analyzed as square-root mean severity (averaged across height within the canopy) and represented as least square means ± SEM. Values of P ≤ 0.05 was considered statistically significant for all models. All analyses were performed using SAS v9.4 (SAS Institute, Cary, NC).

Disease severity for Grove III was 3.32 ± 0.22% and 4.06 ± 0.20% for the 2013–2014 and 2014–2015 citrus seasons, respectively (Table 2). A statistically significant interaction between citrus season and location of fruit (road versus swale side of the tree) was found (Table 3). Multiple comparisons of the interaction revealed significant differences between the severity of fruit symptoms on the road vs. the swale side of the canopy during the 2013–2014 season (Adjusted P = 0.0151); and between the swale side of the canopy in the 2013–2014 season vs. (i) the road side of the canopy (Adjusted P = 0.0159) and (ii) the swale side of the canopy (Adjusted P = 0.0247) during the 2014–2015 season.

### Effect of tree health on CBS incidence and severity in Grove III during the 2014–2015 citrus season

A total of 54 trees were scored as healthy or declining based on tree canopy characteristics during the 2014–2015 citrus season. Thirty-nine were classified as declining and 15 as healthy (Table 4). Health status significantly impacted disease incidence (LSM ± SEM – 72.99 ± 3.25% vs. 59.86 ± 4.52%; P = 0.0202) but not disease severity, the percentage of fruit area covered by symptoms, (P = 0.1884; Table 5). CBS severity was not affected by health status or the side of the tree evaluated (Table 5).

**Table 4:** Health Status: Disease incidence and severity of hard spot lesions on fruit in ‘Valencia’ oranges trees classified as healthy or declining in Grove III during the 2014–2015 citrus season.

Health Status	Side	Height^a^	No. of trees	Fruits with hard spots symptoms	Total no. of fruits	Disease Incidence^b^ (%) LSM ± SEM	Disease Severity^c^ (%) Mean ± Std Dev
Declining			39	1527	1974	72.99 ± 3.25^A^	4.32 ± 4.34
	Road	<1 m		107	131	78.20 ± 5.98	4.71 ± 5.86
		1–2 m		277	328	81.97 ± 3.66	4.82 ± 4.30
		2–3 m		280	357	75.61 ± 3.88	4.33 ± 3.37
		>3 m		173	239	68.60 ± 4.99	3.84 ± 3.90
	Swale	<1 m		95	114	80.41 ± 6.15	3.45 ± 4.19
		1–2 m		175	227	74.01 ± 5.05	5.54 ± 4.54
		2–3 m		257	351	70.71 ± 4.17	4.48 ± 4.26
		>3 m		163	227	66.73 ± 5.23	3.33 ± 3.75
Healthy			15	798	1354	59.86 ± 4.52^B^	3.26 ± 2.96
	Road	<1 m		129	197	68.06 ± 5.98	2.96 ± 2.95
		1–2 m		180	288	64.85 ± 5.10	3.43 ± 2.94
		2–3 m		94	142	69.80 ± 6.48	3.21 ± 3.11
		>3 m		60	92	65.72 ± 7.77	2.94 ± 1.71
	Swale	<1 m		97	204	52.40 ± 6.33	3.21 ± 3.63
		1–2 m		136	242	58.36 ± 5.71	2.53 ± 1.89
		2–3 m		64	111	66.46 ± 7.66	4.28 ± 4.34
		>3 m		38	78	49.88 ± 8.86	3.76 ± 1.71

^a^Height within the canopy. ^b^Disease incidence least square mean values followed by the same letter are not significantly different (P > 0.05, declining vs. healthy only). ^c^Disease severity were based on the severity scale^40^ consisting of six levels, ranging from 0.5% to 49% hard spot coverage on the visible surface (exposed to sunlight) of the fruit and represented here as the calculated mean and standard deviation as indicated by health status, side and height within the canopy.

**Table 5:** Effect of health, side of the tree evaluated and the two-way interactions on incidence and severity of citrus black spot symptoms (hard spots) on ‘Valencia’ oranges in Grove III during the 2014–2015 citrus season.

Fixed Effects	Variables		Disease Incidence (%) LSM ± SEM	Numerator Degrees of Freedom	Denominator Degrees of Freedom	F-Value	Pr > F
**Disease Incidence**							
Side of the tree evaluated	Road		70.23 ± 2.76	1	299.8	10.71	0.0012
	Swale		63.07 ± 3.11				
Health	Declining		72.99 ± 3.25	1	151.2	5.51	0.0202
	Healthy		59.86 ± 4.52				
Height within the canopy	<1 m		68.17 ± 3.69	3	308.2	2.52	0.0583
	1–2 m		69.41 ± 2.96				
	2–3 m		68.23 ± 3.15				
	>3 m		60.87 ± 3.72				
**Fixed Effects**	**Variables**		**Disease Severity**^**a**^ **(%) LSM ± SEM**	**Numerator Degrees of Freedom**	**Denominator Degrees of Freedom**	**F-Value**	**Pr > F**
**Disease Severity**							
Health	Declining		1.94 ± 0.10	1	52	1.78	0.1884
	Healthy		1.70 ± 0.15				
Side of the tree evaluated	Road		1.82 ± 0.11	1	52	0.01	0.9377
	Swale		1.81 ± 0.11				
Health × Side	Declining	Road	1.96 ± 0.11	1	52	0.08	0.7840
		Swale	1.92 ± 0.11				
	Healthy	Road	1.69 ± 0.18				
		Swale	1.71 ± 0.18				

^a^Severity assessments were based on the severity scale consisting of six levels, ranging from 0.5% to 49% hard spot coverage on the visible surface (exposed to sunlight) of the fruit, analyzed as square-root mean severity (averaged across height within the canopy) and represented as least square means ± SEM. Values of P ≤ 0.05 was considered statistically significant for all models. All analyses were performed using SAS v9.4 (SAS Institute, Cary, NC).

### Disease incidence, severity and age

In Grove III a total of 598 fruit were evaluated with an average incidence of 59.03% (range: 0–100%) exhibiting hard spot symptoms within individual tree canopies. The average CBS Severity Index was 31.43% (Range 0–68.06%). In Grove II a total of 4036 fruit were evaluated with an average incidence of 13.68% (Range 0–100%) in the trees surveyed and an average CBS severity index of 4.47% (Range 0–75.0%). Incidence, severity, and CBS Severity Index of hard spot on fruits and the number of fruit in each Grade category for Grove II and III is given in Table 6.

**Table 6:** Age: Groves II and III 2015–2016 citrus season disease incidence and severity of hard spot lesions caused by *Phyllosticta citricarpa* in ‘Valencia’ oranges surveyed in Florida.

Fruit Susceptible Days^a^	Grove	Assessment Date(s)	Age Classification	n	Side	Incidence Positive fruit (%)	Severity Rating Scale^b^ (Number of Fruit)				CBS Severity Index^c^ (%)
							Grade 0	Grade 1	Grade 2	Grade 3	
[2013] 2/17–9/30	II	3/24 and 3/28/2016	Resets	13		4.86	489	24	1	0	1.26
					Road	2.28	257	6	0	0	
					Swale	7.57	232	18	1	0	
			>4 years	76		14.96	2995	374	137	16	4.94
					Road	13.23	1685	186	65	6	
					Swale	17.09	1310	188	72	10	
			All trees	89		13.68	3484	398	138	16	4.47
	III	1/21/2016	>4 years		Road	57.68	146	64	45	90	
					Swale	60.87	99	37	60	57	
			All trees	20		59.03	245	101	105	147	31.43

^a^Fruit Susceptible Days are the date range when fruit are on the tree and are considered susceptible to infection by *Phyllosticta citricarpa* (between fruit set and 24 weeks post fruit set (wpfs)). Dates with year in brackets represent the estimated date of earliest fruit set and latest date of susceptibility at 24 wpfs.

^b^Severity Rating Scale classified fruits within a 1 m^2^ area of the tree canopy based on the number of hard spots on individual fruits; Grade 0 = no hard spots; Grade 1 = 1–5 hard spots; Grade 2 = 5–50 hard spots and Grade 3 ≥ 50 hard spots.

$CBS\,Severity\,Index=\frac{0{n}^{0}+0.25{n}^{1}+0.5{n}^{2}+0.75{n}^{3}}{{n}_{total}}\times 100\,$, where *n*^0^, *n*^1^, *n*^2^ and *n*^3^ represent the number of fruits graded 0, 1, 2 and 3 respectively and *n*_*total*_ the total number of fruits evaluated.

### Effect of grove and canopy side on CBS incidence and severity index during the 2015–2016 citrus season

The incidence and severity (square-root CBS Severity Index) of fruits with hard spot symptoms within a 1 m^2^ area of the canopy for Grove II and Grove III during the 2015–2016 citrus seasons were significantly different (P < 0.0001). There was no effect of side of the tree evaluated on incidence (P = 0.2223) or severity (P = 0.3874) and no interaction between the main effects on incidence (P = 0.6004) or severity (P = 0.6127).

### Effect of tree age and canopy side on CBS incidence and severity index during the 2015–2016 citrus season in Grove II

**Figure 2:**
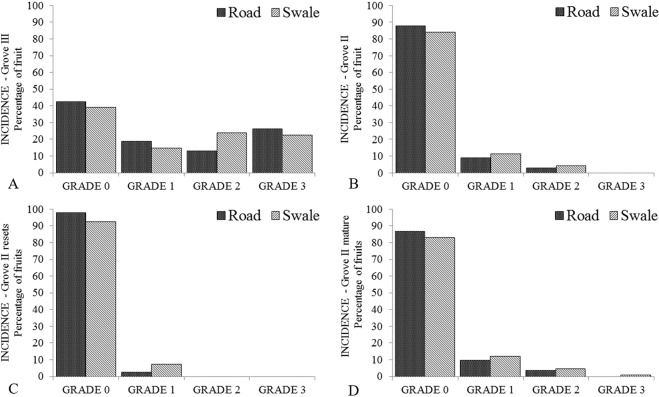
Incidence of Citrus Black Spot on fruit in Grove II and Grove III for the 2015–2016 citrus season. (**A**–**D**) Disease incidence as a percentage of fruits exhibiting hard spots symptoms on either the road or swale side of the tree and graded on a scale 0 to 3 based on severity of the disease on the fruit (Rating 0 = no hard spots; Rating 1 = 1–5 hard spots; Rating 2 = 5–50 hard spots and Grade 3 ≥ 50 hard spots). Disease incidence on fruits in newly fruiting resets (**C**) and in mature citrus trees (**D**).

Of a total of 89 trees evaluated in Grove II, 13 were classified as reset trees that were fruiting for the first time and 76 as mature trees (>4 years) and fruiting during the 2014–2015 citrus season. A significant effect of age (reset vs. mature tree) on disease and severity (P = 0.0066) and a tendency for age to have an effect on incidence (P = 0.0834) was found. However, the interaction between age and side was not significant for either disease incidence or severity (Table 7, Fig. 2). Fruits on resets had a lower incidence and severity of hard spots than fruits in mature trees.

**Table 7:** Effect of age, side of the tree evaluated and the two-way interactions on incidence and severity of citrus black spot symptoms (hard spots) on ‘Valencia’ oranges in Grove II during the 2015–2016 citrus season.

Fixed Effects	Variables		Disease Incidence (%) LSM ± SEM	Numerator Degrees of Freedom	Denominator Degrees of Freedom	F-Value	Pr > F
**Disease Incidence**							
Age	Resets		4.06 ± 3.03	1	98.59	3.06	0.0834
	>4 years		14.48 ± 1.92				
Side of tree evaluated	Road		5.32 ± 2.95	1	121.7	2.43	0.1214
	Swale		11.32 ± 3.33				
Age × Side	Resets	Road	2.11 ± 2.39	1	121.7	1.02	0.3138
		Swale	7.69 ± 4.53				
	>4 years	Road	12.78 ± 2.03				
		Swale	16.36 ± 2.50				
**Fixed Effects**	**Variables**		**Disease Severity**^**a**^ **(%) LSM ± SEM**	**Numerator Degrees of Freedom**	**Denominator Degrees of Freedom**	**F-Value**	**Pr** > **F**
**Disease Severity**							
Age	Resets		0.72 ± 0.48	1	87	7.74	0.0066
	>4 years		2.17 ± 0.20				
Side of tree evaluated	Road		1.20 ± 0.30	1	87	2.82	0.0964
	Swale		1.68 ± 0.30				
Age × Side	Resets	Road	0.36 ± 0.55	1	87	0.66	0.4410
		Swale	1.07 ± 0.55				
	>4 years	Road	2.04 ± 0.23				
		Swale	2.75 ± 0.23				

^a^Severity Rating Scale, fruits were classified based on the number of hard spots on fruit within a 1 m^2^ area of the tree canopy; Grade 0 = no hard spots; Grade 1 = 1–5 hard spots; Grade 2 = 5–50 hard spots and Grade 3 ≥ 50 hard spots.

## Discussion

This is the first study in Florida that examines the disease incidence and severity of CBS on fruit in affected groves since the disease became established in 2010. The incidence and severity of the hard spot lesions on the exposed surface of the fruit within the tree canopy was evaluated in a single grove over a 3-year period and in a second grove during a single citrus season. Grove III had a 100% tree incidence of CBS at the onset of the study and showed a significant increase in incidence of fruits with hard spot lesions from the 2013–2014 to the 2014–2015 citrus seasons. This increase may have been due to the build-up of inoculum in the grove with the high tree incidence of CBS and the conducive weather conditions in 2013 for conidia production and dispersal. In 2013, there were 261 days with temperatures between 15 and 35 °C and RH > 90% for 8 consecutive hours which impacted the 2014–2015 incidence and severity of hard spot lesions on fruit.

It has been reported that hard spot lesions of CBS usually develop on the surface of the fruit exposed to sunlight^23^ and that symptom development is strongly influenced by light^24^. More recent work has shown that hard spot lesions are typically expressed close to color break^25^, a potential effect of ripening that triggers latent infections to develop^26^. In this study, the groves were oriented in a north-south direction, allowing for equal exposure of both the east and west sides of the tree to direct sunlight. If exposure to sunlight is fairly equal on both sides of the tree, one would expect equal expression of the disease on fruit on the road and swale side, however this was not the case. More fruits with lesions were seen on the road side than on the swale side of the tree. Additionally, the severity of the disease was also more pronounced on the fruit found on the road side of the tree during the 2013–2014 citrus season. The ascospore has not been found in Florida to date^18^ and genetic studies also supported this^17,18^, which removes the aspect of wind driven sexual spores from the disease cycle in Florida. This is in contrast to other citrus producing areas where *Phyllosticta citricarpa* is an endemic pathogen on citrus such as in Taiwan^27^, South Africa^2,10^, Australia^9^, and Brazil^28^. More recently, clonal populations of *P. citricarpa* were found in Italy, Malta, and Portugal^8^. The increase in incidence and severity of CBS seen on the road side of trees suggests increased exposure to the conidial inoculum. This could be due to movement of inoculum on vehicle and/or personnel either by direct contact with the spores or as a consequence of the movement of infected plant material.

In the absence of wind-driven ascospores, we investigated the effect of height at which fruits with hard spot lesions were found as a means of assessing conidial dispersal within the tree. In Grove III, there was an interaction on disease incidence between height and season during the 2013–2014 season which was not found in the 2014–2015 season. This could indicate progression of the disease between seasons, with irrigation and rainfall events (with and without high wind) propelling the conidial inoculum upwards and outwards throughout the canopy and downwards due to drip. Weather data conducive to spread of the disease within the canopy, defined as temperature between 15 and 35 °C with relative humidity (RH) ≥ 90% for 8 consecutive hours, were 261 days in 2013. If one considered the presence of susceptible citrus fruit, this was reduced to 160 days. In either scenario, it is conceivable that spread of inoculum within the canopy was possible leading to the progression of the disease from 55% to 68% incidence and the reduction in severity differences between swale and road side of the tree from the 2013–2014 to 2014–2015 citrus seasons (road: 3.7% vs. swale: 2.8% in 2013–2014 to 4.1% vs. 4.0% in 2014–2015 citrus season). The increase in severity on fruit on the swale side of the tree to equal that of the road side of the tree may indicate disease progression from road to swale and indicate the direction from which the inoculum spread. Indeed, it has been shown that wind driven splash droplets can propel conidia upwards and outwards from the point of origin and this distance increases under the influence of wind^16^. Clearly the ascospore is not required for the spread of the disease into the topmost sections of the canopy (>3 m).

An important distinction in the disease cycle in Florida is the absence of the ascospore. The severity on symptomatic fruit was more or less uniformly distributed throughout the canopy during the 2014–2015 citrus season. If the dripping and splashing of conidia in dew, rain, or irrigation water is the sole source of spread of the disease, we would expect that the severity of symptoms on low hanging fruit would be significantly greater than that of fruit higher in the canopy, but this was not the case as equal (2014–2015) or higher (2013–2014) incidence was found in the topmost height of the trees examined. This information, in addition to the lack of the ascospore, paints Kiely^9^ and McOnie (1965) findings in a different light. McOnie concluded that conidia originating from leaf litter was not relevant to the severity of the disease, based on the relative uniform vertical distribution of symptomatic fruits and the observation that fruit located within the splash zone of these leaves did not show an increase in severity. Perhaps in their case the relative uniform vertical distribution of symptomatic fruits was either due to the stage of the disease establishment within the examined groves or a function of both the conidia and ascospore. The current research on the severity and incidence of the disease indicates the significant role of the conidia in disease establishment and spread, confirming work conducted in Brazil^19,28^.

It is intuitive to expect that a healthy plant would perform better than a diseased plant against a pathogen. We examined the impact of other citrus diseases and the general health of citrus trees on the outcome of CBS. Florida citrus has been dealing with the impact of citrus greening (huanglongbing, HLB) since its introduction in 2005^29–31^ and other citrus pathogens such as citrus canker, *Xanthomonas citri*^32^, which together have caused devastating losses to the citrus industry^32,33^. The study of the interaction between these diseases and CBS was not feasible during this study. All trees within this study demonstrated foliar symptoms of HLB, which did not allow for a comparison of trees with and without HLB. Additionally, the incidence and severity of citrus canker was very low in the tree studies (data not shown). However, the overall health of the tree was assessed and classified as either healthy or declining. Purely from the point of symptom expression, declining trees produce less fruit^30^ and thus have less chance for the expression of fruit symptoms on CBS affected trees. Despite this, the data bears out this hypothesis with respect to the development of hard spots on citrus fruit. There was a significant effect on the incidence of CBS: declining citrus trees had a higher incidence of CBS than trees defined as healthy. No effect of tree health on the severity of the disease was found.

In a review of the epidemiology of CBS in South Africa from 1946 to the late 1970s, it was observed that tree condition and age influenced the development of CBS, with older trees being more severely affected than younger trees^10^. This was true in Grove II where reset trees fruiting for the first time had a lower incidence of fruits with hard spot symptoms and lower severity of the disease. A plausible explanation for this is that older trees have been (i) exposed to CBS inoculum for a longer period of time and/or (ii) have more dead or dying canopy material capable of producing inoculum (conidia). Research in Brazil has shown that green twigs colonized by *P. citricarpa* in the grove, once damaged and senescence occurs, produce viable conidial inoculum within 45 days^34^. This implies than any activity that leads to senescence of infected tissue, whether physiological or pathological, can lead to the release of viable inoculum within 45 days, provided that the climatic conditions are conducive. If temperature (15–35 °C) and moisture (RH ≥ 90% or rainfall >0.25 mm) conditions in Florida are conducive for tree tissue infection at least one-third of the year, the disease will continue to spread throughout infected groves into clean citrus areas and continually pose a problem on citrus fruit. A more comprehensive look at the reduction of the inoculum year-round is required to control the disease. A steady buildup of inoculum within the canopy of the citrus tree will continue to occur in Florida if steps are not taken to manage this disease year-round.

Fungicide active ingredients currently recommended and labeled for the control of CBS in Florida are copper, strobilurins, fenbuconazole, and premix combinations, such as azoxystrobin/difenoconazole and pyraclostrobin/boscalid. Recommendations entail the application of these fungicides on a 21–28 day cycle from early May to mid-September^35^ depending on the citrus variety, spanning approximately 130 days. Recent research suggests that extending the application time to protect the fruit to 180 or 220 days^36^ would improve control of the disease, reducing incidence and severity of CBS symptoms by up to 96% and crop loss due to premature fruit drop by up to 77%. Extending the fungicide application period would better control the continuous conidia inoculum within the canopy and secondary inoculum on fruits in Florida and potentially slow the spread of the disease while other modes of control are developed.

## Methods

### Data collection

For the purpose of this study, disease incidence is defined as the number of CBS positive fruit (hard spot symptoms)/total number of fruits examined, and disease severity is defined as the percentage of fruit surface covered by hard spot lesions. The disease incidence and severity of CBS was examined over a 3-year period (2013 to 2016) in three commercial citrus groves in Florida. The groves are organized with irrigation ditches (swales) and roads (drivable surface) alternating between rows of citrus (row-swale-row-road-row-swale), and citrus rows were oriented in roughly a north-south direction in each grove. The cultivar-rootstock combination was ‘Valencia’ grafted onto Swingle, planted on average 3.36 m (Grove II) and 3.33 m (Grove III) apart within rows and 6.70 m between rows. Tree age varied from resets (1.5–2 years, with or without fruit) to mature trees (>4 years and fruiting). Initial plot maps were prepared and findings on the spatial and temporal distribution of CBS on fruit in Grove II and another grove not discussed in this paper (Grove I) has been previously published^37^. The data on the incidence and severity of CBS on fruit in the canopy was taken from Grove II and III.

The study area in Grove II consisted of 37 rows of citrus containing 124 to 127 citrus trees per row. During the 2015–2016 citrus season, there were 4339 trees with 167 open spaces (no tree) and 26.57% (1153/4339) were classified as positive for CBS. The study area in Grove III was comprised of 27 rows of citrus containing 98 to 111 citrus trees per row. In 2013–2014 citrus season, there were 2849 tree spaces, with 1659 trees and 1190 open spaces and 100% were positive for CBS.

### Weather data

The Florida Automated Weather Network’s (FAWN) Immokalee station, located at 26°27′43.5″N 81°26′25.9″W is approximately 11.08 km SSE from Grove II and 25.92 km WNW from Grove III and was used to gather 15 minute data on soil temperature, air temperature at 0.6, 2 and 10 m, relative humidity (RH) at 2 m (%), dew point (DP) at 2 m, rainfall, wind speed at 10 m and solar radiation between 1 Jan 2010 and 15 Jun 2017. This station was chosen due to its close proximity to the groves and availability of archived data. A 24-hr period, rainy day, and wet canopy were defined as previously described^37^. Briefly, a 24-hr period started at 12:00 am and ended at 11:59 pm; a rainy day had a total daily rainfall (TDR) greater than or equal to 0.25 mm; and a wet canopy was a day in which RH was greater than or equal to 90% for at least 8 consecutive hours^38^. A secondary definition of a wet canopy was defined as a day when the dew point, DP, the temperature at which water vapor in the air condenses, was greater than the temperature (T) at 0.6 m. Data used to estimate susceptibility period of fruit within the canopy to CBS were generated as described in Hendricks *et al*.^37^, however fruit susceptibility was calculated as occurring in the first 168 days (24 weeks) following fruit set (wpfs) according to work done by Baldassari *et al*.^13^ on ‘Valencia’ and ‘Natal’ orange varieties. To date there is no evidence to suggest that the sexual spore (ascospore) is present in Florida^17,18,39^, hence only published literature values relevant to the conidia were used to determine susceptibility of tree tissues, including fruit. Conservatively, the temperature range selected was 15 to 35 °C, allowing for the production and germination of conidia. Susceptibility of tree tissues was considered throughout the entire year and defined as periods in which temperature and moisture were sufficient for infection based on literature values for the conidia. For T, RH and DP, a minimum period of 8 consecutive hours was required to be included in the data used to develop the ten categories for potential infection period (IN). These categories were designated as IN1 to IN5 for fruit and IN6 to IN10 for other citrus tissue. These were defined as:

FRUIT SUSCEPTABILITY (IN1 to IN5)

1 $${\rm{IN}}1={{\rm{T}}}_{15\mbox{--}{35}^{\circ }{\rm{C}}}+{\rm{DP}}\ge {\rm{Tmin}}\,{\rm{for}}\,8\,{\rm{hr}}+{\rm{Susceptible}}\,{\rm{Fruit}}$$

2 $${\rm{IN}}2={{\rm{T}}}_{15\mbox{--}{35}^{\circ }{\rm{C}}}+{\rm{RH}}\ge 90 \% \,{\rm{for}}\,8\,{\rm{hr}}+{\rm{Susceptible}}\,{\rm{Fruit}}$$

3 $${\rm{IN}}3={{\rm{T}}}_{15\mbox{--}{35}^{\circ }{\rm{C}}}+{\rm{TDR}}\ge 0.25\,{\rm{mm}}+{\rm{Susceptible}}\,{\rm{Fruit}}$$

4 $${\rm{IN}}4={{\rm{T}}}_{15\mbox{--}{35}^{\circ }{\rm{C}}}+{\rm{RH}}\ge 90 \% \,{\rm{for}}\,8\,{\rm{hr}}+{\rm{TDR}}\ge 0.25\,{\rm{mm}}+{\rm{Susceptible}}\,{\rm{Fruit}}$$

5 $${\rm{IN}}5={{\rm{T}}}_{15\mbox{--}{35}^{\circ }{\rm{C}}}+{\rm{RH}}\ge 90 \% \,{\rm{for}}\,8\,{\rm{hr}}+{\rm{TDR}}\ge 0.25\,{\rm{mm}}+{\rm{DP}}\le {\rm{T}}\,{\rm{for}}\,{\rm{8hr}}+{\rm{Susceptible}}\,{\rm{Fruit}}$$

TREE TISSUE SUSCEPTABILITY (IN6 to IN10)

6 $${\rm{IN}}6={{\rm{T}}}_{15\mbox{--}{35}^{\circ }{\rm{C}}}+{\rm{DP}}\ge {\rm{Tmin}}\,{\rm{for}}\,8\,{\rm{hr}}$$

7 $${\rm{IN}}7={{\rm{T}}}_{15\mbox{--}{35}^{\circ }{\rm{C}}}+{\rm{RH}}\ge 90 \% \,{\rm{for}}\,8\,{\rm{hr}}$$

8 $${\rm{IN}}8={\rm{T}}15\mbox{--}{35}^{\circ }{\rm{C}}+{\rm{TDR}}\ge 0.25\,{\rm{mm}}$$

9 $${\rm{IN}}9={{\rm{T}}}_{15\mbox{--}{35}^{\circ }{\rm{C}}}+{\rm{RH}}\ge 90 \% \,{\rm{for}}\,8\,{\rm{hr}}+{\rm{TDR}}\ge 0.25\,{\rm{mm}}$$

10 $${\rm{IN}}10={{\rm{T}}}_{15\mbox{--}{35}^{\circ }{\rm{C}}}+{\rm{RH}}\ge 90 \% \,{\rm{for}}\,8\,{\rm{hr}}+{\rm{TDR}}\ge 0.25\,{\rm{mm}}+{\rm{DP}}\le {\rm{T}}\,{\rm{for}}\,8\,{\rm{hr}}$$

If there was a 24-hr period in which the conditions for infection were met, that day counted towards the susceptibility data.

### 2013–2015 disease incidence and severity of citrus black spot on fruit

During the 2013–2014 and 2014–2015 citrus seasons, two trees from each row of Grove III, totaling 54 trees, were randomly chosen for the assessment of CBS incidence and severity on fruit. The road and swale side of a tree was assessed at four heights (<1 m, 1–2 m, 2–3 m and >3 m), and fruits were evaluated within a 1 m^2^ section at each height. Severity assessments were based on a rating system devised by Sposito *et al*.^40^; ratings ranged from 0.5% to 49% hard spot coverage on the visible surface of each fruit^28,40^. The mean severity was based on an average of all the positive fruits evaluated within a 1 m^2^ section of the canopy. For example, if there were 20 fruits in a 1 m^2^ sample area and 5 were positive, each with a severity rating of 1.5%, the mean severity rating at that height was 1.5%. Additionally, disease severity and incidence for the entire tree (combining road and swale side) was calculated from this data. During 2014–2015, trees examined were classified as either healthy or declining. Citrus were defined as declining if they exhibited a combination of the following canopy features: sectored within the canopy or as a whole, thinning; reduced growth flushes, die back, reduced fruiting and/or vegetative sprouts on the trunk or on larger interior branches.

### CBS severity index

During the 2015–2016 citrus season, a severity rating for Grove III was done on fruit from 20 randomly selected trees, two from each row. Additionally, 89 randomly selected CBS positive trees in Grove II were evaluated for CBS severity. Fruit within a single 1 m^2^ sampling area taken on the swale and road sides of the tree were graded based on the number of lesions on the fruit using a 0 to 3 grading system. Fruits with no lesions were given a grade of 0; fruits with 1–5 hard spots were given a grade of 1; fruits with 6–50 spots were given a grade of 2 and grade 3 was reserved for fruits having greater than 50 spots.

The CBS severity index was calculated based on the following equation:

11 $$CBS\,Severity\,Index=\frac{0{n}^{0}+0.25{n}^{1}+0.5{n}^{2}+0.75{n}^{3}}{{n}_{total}}\times 100\,$$

where *n*^0^, *n*^1^, *n*^2^ and *n*^3^ represent the number of fruits graded 0, 1, 2 and 3 respectively and *n*_*total*_ the total number of fruits evaluated.

### Statistical analysis

#### Effect of citrus season and fruit location (height and side) within the canopy on CBS incidence and severity in Grove III

The data on disease incidence were fit to a generalized linear mixed model assuming a binomial distribution with a repeated measures effect of tree based on compound symmetry. Disease incidence was modelled as a function of the citrus season (2013–2014 and 2014–2015), side of the tree evaluated (swale or road), height within the canopy at which diseased fruit was found (<1, 1–2, 2–3, <3 m) and the interactions between these factors. Non-significant effects were removed hierarchically to obtain the final model of citrus season, side of the tree evaluated, height within the canopy, and the interaction between citrus season and height.

Mean severity was calculated for each side of the tree by averaging the observed percentage for the four heights within the canopy. These data were transformed by square-root to obtain approximate normality of the residuals. Square-root mean severity was modelled as a function of the fixed effects of citrus season (2013–2014 and 2014–2015), side of the tree evaluated (swale or road), and the interactions between these factors and a random effect for the repeated measures on trees.

#### Effect of health on CBS incidence and severity in Grove III during the 2014–2015 citrus season

The data on disease incidence were fit to a generalized linear mixed model assuming a binomial distribution with a repeated measures effect of tree using compound symmetry. Disease incidence was modelled as a function of the health, side of the tree evaluated (swale or road), height within the canopy at which diseased fruit was found (<1, 1–2, 2–3, >3 m) and the interactions between these factors. Non-significant effects were removed hierarchically to obtain the final model of health, side of the tree evaluated, and height within the canopy.

Mean severity was calculated as described above and transformed by square-root to obtain approximate normality of the residuals. Square-root mean severity was modelled as a function of the fixed effects of health, side of the tree evaluated (swale or road), and the interactions between these factors and a random effect for the repeated measures on trees.

#### Effect of grove and tree side on CBS incidence and severity index during the 2015–2016 citrus season

Incidence of CBS affected fruit within 1 m^2^ area of the canopy was calculated for each side of the tree based on grading data (Incidence = (Grade 1 + Grade 2 + Grade 3)/(Grade 0 + Grade 1 + Grade 2 + Grade 3)). Incidence was fit to a generalized linear mixed model assuming a binomial distribution and modelled as a function of the fixed effect of grove, side of the tree evaluated (swale or road) and their interaction and the random effect of tree.

Disease severity was assessed based on a CBS severity index adapted by Truter (2010)^41^ from previous work by De Wet (1987). These data were transformed by square-root to obtain approximate normality of the residuals. Square-root CBS Severity Index was fit to a generalized linear mixed model with a repeated measures effect of tree assuming a normal distribution and modelled as a function of grove, side of the tree evaluated (swale or road) and the interaction.

#### Effect of tree age on CBS incidence and severity index during the 2015–2016 citrus season in Grove II

A subset of data from the 2015–2016 season taken solely from Grove II containing tree age was used to model the effect of age on the incidence of CBS affected fruit and on CBS Severity Index. Disease incidence (Incidence = (Grade 1 + Grade 2 + Grade 3)/(Grade 0 + Grade 1 + Grade 2 + Grade 3)) were fit to a generalized linear mixed model assuming a binomial distribution and modelled as a function of age, side of the tree evaluated (swale or road) and their interactions.

As above, CBS severity index data were transformed by square-root to obtain approximate normality of the residuals and the data were fit to a generalized linear mixed model. Square-root CBS Severity Index was modelled as a function of age, side of the tree evaluated (swale or road) and their interactions. Square-root CBS Severity Index was modelled as a normal distribution random variable.

Values of P ≤ 0.05 was considered statistically significant for all models. All analyses were performed using SAS v9.4 (SAS Institute, Cary, NC).

## Supplementary information

Supplementary information.

## Data Availability

The datasets generated during and/or analyzed during the current study are available from the corresponding author on reasonable request.
