# Supplementary material for: The Effect of Weather and Location of Fruit within the Tree on the Incidence and Severity of Citrus Black Spot on Fruit
Source: Sci Rep. 2020 Jan 29;10:1389. doi: 10.1038/s41598-020-58188-z (PMC6989463; doi:10.1038/s41598-020-58188-z)
Supplement: Supplementary file 1 — Supplementary information. [file 41598_2020_58188_MOESM1_ESM.pdf]

The Effect of Weather and Location of Fruit within the Tree on the Incidence and Severity of Citrus Black Spot on Fruit

Katherine E. Hendricks[1,+], **Mary C. Christman**[2,+], **and Pamela D. Roberts**[1,+*]

[1]University of Florida, IFAS-Southwest Florida Research and Education Center, Department of Plant Pathology, Immokalee FL 34142, USA
[2]University of Florida, Department of Statistics, Gainesville, Florida, 32611, USA
[*]pdr@ufl.edu
[+]these authors contributed equally to this work

Supplementary Figures

Figure S1. Fruit symptoms. A. Hard spots or shot hole spots (arrows) are small, round, sunken lesions with brick-red to chocolate brown margins and gray centers. Hard spot lesion may also have a green halo. Inset. Hard spots with fungal structures (pycnidia) in the center; fungal structures appear as elevated black dots in the center of lesions. B. Freckle or early virulent spots (arrow heads) are small irregularly shaped reddish lesions. False melanoses appear as numerous small, slightly raised tan to dark brown lesions. Virulent spots occur when multiple lesions coalesce typically in heavily infected fruit, giving it a leathery appearance.

Supplementary Tables

Table S1. Grove III. Count data used in the analysis of disease incidence of hard spot lesions in the canopy during the 2013-2014 and 2014-2015 citrus season caused by *Phyllosticta citricarpa* in 'Valencia' oranges surveyed in Florida.

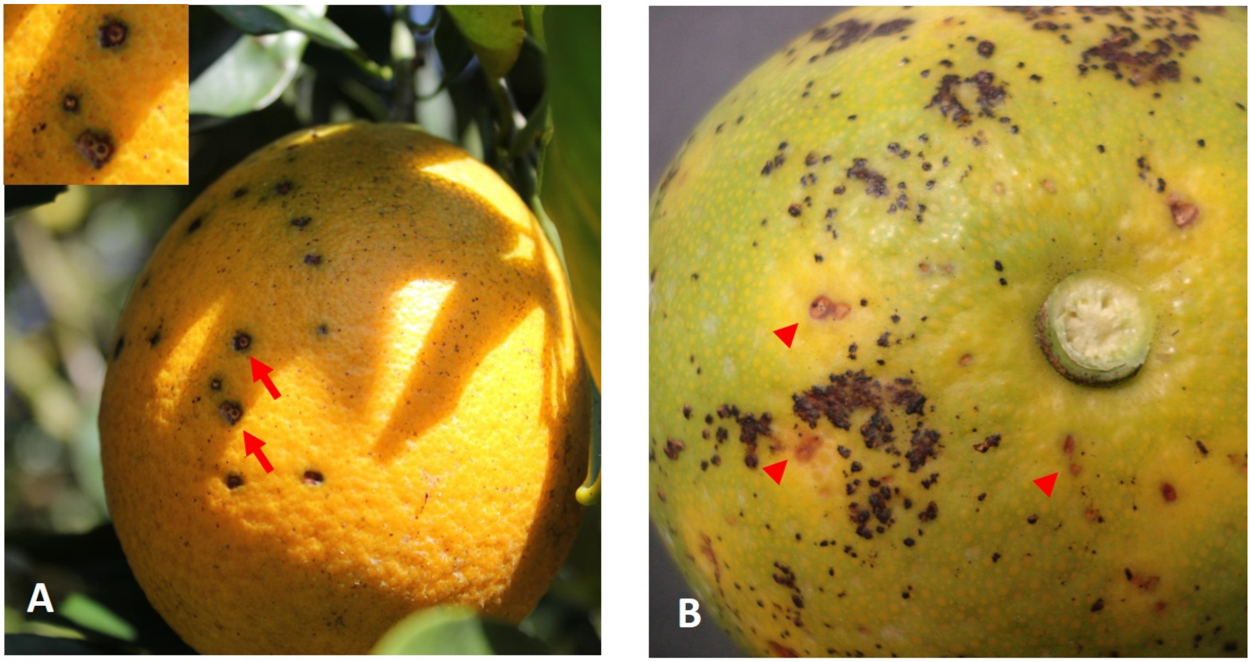

Figure S1. Fruit symptoms. A. Hard spots or shot hole spots (arrows) are small, round, sunken lesions with brick-red to chocolate brown margins and gray centers. Hard spot lesion may also have a green halo. Inset. Hard spots with fungal structures (pycnidia) in the center; fungal structures appear as elevated black dots in the center of lesions. B. Freckle or early virulent spots (arrow heads) are small irregularly shaped reddish lesions. False melanoses appear as numerous small, slightly raised tan to dark brown lesions. Virulent spots occur when multiple lesions coalesce typically in heavily infected fruit, giving it a leathery appearance.

Table S1. Grove III. Count data used in the analysis of disease incidence of hard spot lesions in the canopy during the 2013-2014 and 2014-2015 citrus season caused by *Phyllosticta citricarpa* in 'Valencia' oranges surveyed in Florida.

Citrus Season	Fruit Susceptible Days[1]	Assessment Date	Trees	Level	Total fruits[2]	Fruits with Hard Spot Lesions			Road side[4]		Swale[5]	
						Swale	Road	Tree[3]	Positive	Total	Positive	Total
2013-2014	[2013] 2/6 – 9/25	2/13 and 2/18/2014	54	Total	4340	886	1437	2323	1437	2537	886	1803
				< 1m	970	178	285	463	285	568	178	402
				1-2 m	1606	355	463	818	463	929	355	677
				2-3 m	1468	353	445	798	445	744	353	724
				>3 m	296	0	244	244	244	296	0	0
2014-2015	[2014] 3/29 – 10/30	3/31 and 4/1/2015	54	Total	3328	1025	1300	2325	1300	1774	1025	1554
				< 1m	646	192	236	428	236	328	192	318
				1-2 m	1085	311	457	768	457	616	311	469
				2-3 m	961	321	374	695	374	499	321	462
				>3 m	636	201	233	434	233	331	201	305

[1]Fruit Susceptible Days are the number of days when fruit are on the tree and are considered susceptible to infection by *Phyllosticta citricarpa* (between fruit set and 24 weeks post fruit set (wpfs)). Dates with year in brackets represent the estimated date of earliest fruit set and latest date of susceptibility at 24 wpfs.

[2]Total number of fruits examined for hard spots symptoms in a 1m^2 area evaluated in total or at the level indicated within the canopy of the 54 trees examined.

[3]Total number of fruits having hard spots in a 1m^2 area evaluated in total or at the level indicated within the canopy of the 54 trees examined.

[4]Count data for fruits with hard spots evaluate within a 1m^2 section of the canopy and at each level and in total as seen from the road.

[5]Count data for fruits with hard spots evaluate within a 1m^2 section of the canopy and at each level and in total as seen from the swale.
